# Exploring the Social Networks’ Use in the Health-Care Industry: A Multi-Level Analysis

**DOI:** 10.3390/ijerph18147295

**Published:** 2021-07-08

**Authors:** Tommasina Pianese, Patrizia Belfiore

**Affiliations:** 1 Institute for Studies on the Mediterranean, National Research Council of Italy (CNR ISMed), 80132 Napoli, Italy; 2 Department of Movement and Wellness Sciences, Parthenope University of Naples, 80132 Napoli, Italy; patrizia.belfiore@uniparthenope.it

**Keywords:** social media, social network, healthcare, literature review, e-health, technology

## Abstract

The application of social networks in the health domain has become increasingly prevalent. They are web-based technologies which bring together a group of people and health-care providers having in common health-related interests, who share text, image, video and audio contents and interact with each other. This explains the increasing amount of attention paid to this topic by researchers who have investigated a variety of issues dealing with the specific applications in the health-care industry. The aim of this study is to systematize this fragmented body of literature, and provide a comprehensive and multi-level overview of the studies that has been carried out to date on social network uses in healthcare, taking into account the great level of diversity that characterizes this industry. To this end, we conduct a scoping review enabling to identify the major research streams, whose aggregate knowledge are discussed according to three levels of analysis that reflect the viewpoints of the major actors using social networks for health-care purposes, i.e., governments, health-care providers (including health-care organizations and professionals) and social networks’ users (including ill patients and general public). We conclude by proposing directions for future research.

## 1. Introduction

Social media are internet-based tools through which individuals, organizations and communities share, exchange or co-create information, ideas, images and other contents within a virtual network [1,2]. They include blogs, social networks, video and photo-sharing sites, wikis and other social media which can be grouped in social and professional networking (e.g., Facebook, LinkedIn), media sharing (e.g., YouTube), content production as blogs and microblogs (e.g., Tumblr), knowledge information aggregation (e.g., Wikipedia), virtual reality and gaming (e.g., Second life) [2,3]. All these web-based technologies and services are centered around social interactions and put emphasis on enabling users to communicate, interact, edit, and share contents in an online environment. They are designed as a dialogue among geographically dispersed users who can produce valuable information resources, solve challenging problems by tapping into unique and rare expertise, and gain diverse insights and perspectives.

The application of social media has become increasingly prevalent in the health domain, because it offers a platform to the public, patients, and health-care providers to communicate about health issues with the possibility of potentially improving health outcomes [4]. Their functionalities vary from the promotion of health programs and patient education to professional networking until the use by hospitals, pharmaceutical companies, etc. to communicate with the community and patients, enhancing visibility, and marketing products and services [2].

In this study, we focus our attention on social networks—that is, web-based technologies which bring together a group of people and health-care providers that have health-related interests in common; who share text, image, video and audio content; and interact with each other. They include open social networks (e.g., Facebook, Linkedin, Twitter, Instagram) which have not been designed for health-related interactions, as well as intentionally designed social networks (e.g., PatientsLikeMe, Mumsnet, TreatmentAction Campaign, My Pro Ana) with an explicit interest in health and health behaviors [5]. Whether they are open or intentionally designed social networks, online communities are likely to increase knowledge about health and even influence the health status of people [5,6,7,8]. For example, it has been demonstrated that social ties in the virtual environment reduce risks of depression among the elderly and boosts their self-confidence [9]. Likewise, Naslund et al. (2016) have focused on the importance of online peer-to-peer support for patients with chronic disease, cancer, rare diseases, maternal and infant care, depression, etc. [1] Social networks have played a role also during the COVID-19 pandemic that started in the early 2020s. In an emerging and spontaneous way, they have become an important channel for disseminating information on the appropriate behaviours to avoid contagion, to support patients and their families, as well as for sharing information on a poorly understood virus, crucial for identifying real-time responses to the crisis in progress [10]. The downside has been that social networks have become tools for the dissemination of false information, not supported by scientific evidence.

The increasing relevance of social networks in healthcare explains the growing attention of scholars and researchers. Despite the growing number of studies on this topic, efforts to map the overall research on social networks’ use in healthcare have so far been rather limited. In this regard, Moorhead et al. (2013) have provided an overview of the benefits of social media for health communication [4]. Househ et al. (2014) and more recently Zhao and Zhang (2017) have conducted literature reviews aimed at understanding the users’ health information seeking behaviours on social networks [11,12]. These reviews focus on the patients’ perspective and thus offer a partial view of the phenomenon. We contribute to this debate by providing a broader and multi-level overview of the studies that has been carried out to date on social network uses in healthcare, taking into account the great level of diversity that characterizes the health-care industry [13]. To this end, we conduct a scoping review enabling to understand how research on social network use in healthcare has progressed over time and identify the major research streams [14,15]. Then, we aggregate knowledge according to three levels that reflect the viewpoints of the major actors using social networks for health-care purposes, i.e., governments, health-care providers (including health-care organizations and professionals) and social networks’ users (including ill patients and general public).

The article is organized in the following way. First, we present the research method followed for this scoping review of literature that has addressed the social network use in healthcare. Then, we aggregate and report knowledge from reviewed studies to the three over mentioned levels. Last section offers conclusive remarks and propose directions for future research.

## 2. Materials and Method

We have undertaken a scoping review, which is an approach to literature review designed to provide an overview of a complex research area [14,15]. The aim of a scoping review is to map the literature on a particular topic, identify key concepts along with gap in existing research [14,15]. By looking at how research on social network use in healthcare has progressed over time, this scoping review enables us to identify the major research streams, synthesize the state of knowledge and define a research agenda [16,17,18,19,20,21].

In the following, we describe the method and stages that, in line with the PRISMA (Preferred Reporting Items for Systematic Reviews and Meta-Analyses), have been designed to select and analyse studies included in this scoping review [22]. As for document search, we have retrieved documents published between January 2004 and December 2020 in the Elsevier’s Scopus, Web of Science and Google Scholar databases which together include the main peer-reviewed literature for economics and business research [23]. Although we recognize the importance of contemporary findings provided by grey literature, we have excluded it because we are interested in methodologically rigorous studies published in referred journals and books. We have chosen 2004 as a starting date for our search because social networks began seriously to spread following the launch of Facebook in 2004 in the United States, which is currently the leading social network in the world for the number of active users.

Coherently the methodological principles of the scoping review, the broad keywords “social network” and “healthcare” have been used in combination for search on title, abstract and/or keywords of English-written documents (journal articles and book chapters) in selected databases [24].

The search on selected databases has yielded n. 93 documents on Elsevier’s Scopus (subject area business and management), n. 583 documents on Web of Science. We have used the search string “social network and healthcare” in Google Scholar, which has resulted in 59 documents. All titles of these documents have been reviewed for relevance and eligibility. From this initial sample, we have excluded duplications and documents on not pertinent field of investigation (e.g., health services issues). Over 450 documents have been selected whose references have been checked to identify additional studies that may have been missed in the initial search. This has yielded n. 7 other documents.

**Figure 1:**
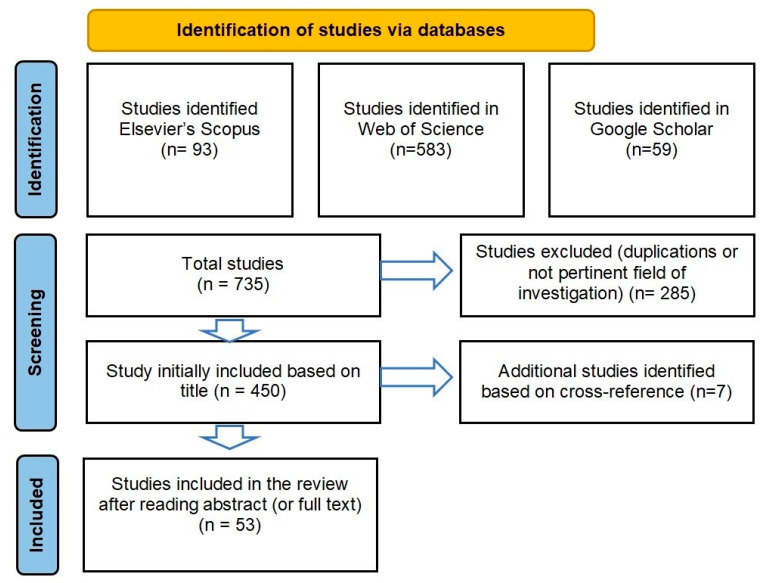
Flow diagram of the reporting items from PRISMA. Source: adapted for PRISMA.

Following the thorough reading of abstracts (or full texts when necessary), we have considered eligible for inclusion studies that meet the following criteria: (1) full-length articles or reviews; (2) social network not referred to the Social Network Analysis (SNA) intended as a process for studying the features of the social structures; (3) use of social media and specifically social network for health-related purposes by health organizations, professionals and users. Authors independently reviewed the studies and reached consensus on the inclusion for the analysis. Through these procedures, 53 documents have been identified as shown in the following Figure 1. Although this sample may not be exhaustive, it is believed that the reviewed documents comprise a reasonably representative body of the research accomplished on the topic under investigation.

As for data analysis, we have drawn on principles of thematic coding to derive themes from the data, where our “data” are the reviewed documents [25,26]. Themes represent the fundamental arguments and conceptual linking of concepts and core ideas addressed by each author(s). We have not extracted themes from their context, rather we have inducted themes from our holistic understanding of each study.

An inductive process of theme identification has been followed, i.e., a systematic process of interpretative synthesis which is an alternative to the deductive application of a pre-determined analytical framework and/or conceptual categories [27]. The iterative process of theme identification and checks for consistency and validity has been detailed, thus resulting at first in a considerable number of finely grained themes. Then, they have been refined, classified and synthesized to identify “super-themes” under which “sub-themes” are hierarchically arranged after extensive checks for duplication and redundancy [26]. Iteration continued until we arrived at major themes which have been grouped and following discussed according to three levels reflecting the viewpoints of distinct actors using social networks, i.e., governments, health-care providers (including health-care organizations and professionals) and social networks’ users (including patients and not ill patients using social networks for health-related purposes). The following Table 1 displays the distribution of reviewed documents by levels of analysis, along with main themes and sub-themes addressed for each. 

### Descriptive Analysis of Reviewed Studies

As for the progression of research over time, we have divided the timeframe 2004–2021 in two distinct periods. Thirteen studies (24%) have been published in the first period (2004–2012), while a growing interest toward social network use in healthcare is evident between 2013 and 2021 with the publication of 40 of the reviewed studies (76%). This is in line with a more general attention given in the last decade by researchers to a variety of interdisciplinary aspects of social networks following to their diffusion in everyday life. In this regard, it is worth noting that most of the studies on implications of social networks in healthcare appear on health-related academic journals (60%), with regard, e.g., to the role of virtual communities for patients or the Facebook use for advertising weight-loss products. The remaining part has been published in journals centred on management and Information Systems (e.g., use of social networks by physicians with self-promoting purposes), and public administrations (e.g., opportunity for governments to obtain real-time data through social networks).

As for their nature, 37% are theoretical/conceptual studies (i.e., 20 works). These studies are weakly theorized, with most conceptual studies having no theoretical foundation excepted for Burton-Jones et al. (2020) and Munson et al. (2013) who referred, respectively, to Institutional theory and Socio-technical theory to explain the process of diffusion of social networks in healthcare [28,29]. Likewise, only two studies have developed conceptual frameworks referred to the knowledge sharing among physicians in virtual environments and peer-to-peer support through social media [1,30]. The majority of studies limit to the anecdotal description of the phenomenon (e.g., benefits and risks following the use of social networks) or providing with guidelines for effective digital communication actions (e.g., definition of social media plan in hospitals for effective marketing strategies).

In terms of methodology employed in the remaining 33 empirical studies (i.e., 63% of our sample), the majority has based their investigations on the administration of surveys to social network’s users (see the following Table 2). Many researchers have referred to data from social network pages/accounts, thus confirming the key-role of these technological platforms in providing with real-time data for the accurate analysis and understanding of dynamics in the healthcare industry [5].

## 3. A Multi-Level Analysis of Studies Dealing with Social Network Use in Healthcare

Discussion of studies under review has been grouped according to the emphasis put on governments, health-care providers and social network users. The first describes the opportunities and challenges faced by governments because of the e-health and care management. The second focuses on the implications of social network use by (a) health-care organizations (HCO), intended as purposefully designed, structured social system developed for the delivery of health-care services by specialized workforces to defined communities; (b) health-care professionals, including individuals with specialized skills and knowledge, gained through formal training and experience, which provide health-care treatment and advice. The third refers to motivations, concerns, as well as the practices and appropriation of social networks by patients, i.e., a person who is receiving medical care or who is cared by a particular doctor, as well as by people that—even if not sick—may use social networks for health-related purposes. In the following, we discuss each of them in detail.

### 3.1. Governments

Among the reviewed studies, there is a recognition of the opportunities for institutions following the use of social networks for health-care purposes. These encompasses the opportunities to reduce social disparities [31,32,33], exert pharmacovigilance by exploiting real-time data [34,35], support people with mental disorders [36], and promote campaigns for healthy behaviours [5,7].

Some authors argue that social networks may be exploited to reduce social inequalities [32,33] and rural-urban health disparities [31]. Health-focused social networking sites (e.g., PatientsLikeMe, Mumsnet) have the potential to link people who have a health experience in common and who would otherwise not interact because they are geographically isolated from each other, because they are limited in their ability to interact socially or because interactions about their health conditions are stigmatized [32]. Patients may use social networks to amplify their voices and make pressure on relevant actors through supporting campaigns aimed to direct policies and practices toward issues critical to their pathologies (e.g., funding research on rare genetic diseases) [33], and even get to change health systems [32]. Likewise, by analysing the patterns of communications exchanged among users of online health communities, Goh et al. (2016) has found that social networks are likely to be instruments of social equity because people living in urban areas support those who live in rural areas by sharing health knowledge and increasing their capabilities, thereby alleviating disparities between urban and rural areas, which sometimes is significant [31].

Social networks are also viewed as powerful tools for bio-surveillance by several studies [34,35,36]. People use social networks to share with other users, personal experiences and opinions on medical conditions, therapies and side effects, which often they are reluctant to discuss with health-care workers. This real-time data is collected faster than clinical data and, if pre-processed (i.e., cleaned from useless and noisy text) and then analysed with appropriate techniques and software, enable a surveillance capability for governments [34]. This refers to the monitoring of health-damaging behaviours (e.g., smoking, drugs and alcohol use), as well as to self-medication actions and side effects of medicines [34,35]. According to Moreno et al. (2011), Facebook can be used also as an innovative way to identify young people with mental health disorders or struggling with depression [36].

Despite recognizing their potentialities, another group of studies have pointed at the challenges that should be managed by institutions when considering the social network uses for health-related purposes [29,37,38,39]. 

First, the digital divide, i.e., geographical areas have different access to technologies and/or people have different technology-related knowledge, remains a priority issue to be solved if governments intend to focus on technological innovations to improve the health systems [40]. Second, open social networks (e.g., Facebook) are important for governments to influence people to embrace healthy lifestyles and to understand the population sentiment about, e.g., vaccine and the behaviours that people are willing to engage in [5,7]. However, they are also channels for the spread of false, or incomplete health information that lack of scientific evidence, which has the potential to compromise governments’ activities and image, as well as undermine progress in medicine and healthcare [41]. Misinformation is exacerbated by echo chamber effect—i.e., misinformation is amplified when network increases, as well as by social manipulations which may result from individual account activities (“social bots”) and/or “inauthentic behaviors” referred to activities deliberately put in place to artificially manipulate the conversations to make them appear more popular than they are [37]. Third, governments have an incumbent obligation to intervene to ensure the balance among privacy (i.e., right to decide which, how and when communicate personal information), security (i.e., confidentiality, prevent disclosure personal information to unauthorized individuals) and sharing personal data on social networks to ensure that the disclosure of sensitive health information does not harm the person and his or her family and friends [29,39]. Studies have found that many people release personal information on social networks because they do not realize the risks associated with it, nor do they know what tools are available to protect themselves and which privacy policies are in place. They feel a social pressure to be present on social networks and share information assuming incorrectly they interact only with their closest contacts [39]. The literature has addressed the privacy issue and underlined that the designers of social networks should help people configure their sharing setting appropriately [29]. Likewise, Lau et al. (2012) put in evidence the need that governments establish a regulatory body and policies ensuring that social media are used in a safe and effective manner, to educate users’ skills in order to improve their attitude to social media (safe access and/or production of contents and materials); to develop a scientific approach in design the public health-related campaigns in order that people can really understand the message sent by government and public bodies [38].

### 3.2. Health-Care Providers

#### 3.2.1. Health-Care Organizations

Shifting the perspective to the organisational level, another group of studies has analyzed the drivers and barriers to the adoption of social networks by health-care organizations, as well as attempted to provide with principles and guidelines for the elaboration of an effective plan for strategic digital communication.

Social networks represent an effective and costless tool for health-care organizations to communicate with patients, having huge potential for their engagement through institutional as well as informal posts, photos, and videos sharing [42]. They offer room for an interactive environment where clinics may communicate in real-time with patients, getting them engaged in ongoing dialogues beyond doctor consultations that allow healthcare information to be provided, treatments to be followed up and questions to be answered, making processes more efficient and customer oriented, and facilitating the development of closer relationships with patients [32,43,44]. Health messaging may also be acted on by the patients, thereby opening a dialogue with the organization that allows both parties to work collaboratively to address issues affecting the health and well-being of the social networks’ users [42]. These digital platforms also offer the opportunity to provide information about the organizations, to inform and keep the public up to date with themes of public interest, as well as to report about internal research activities [45].

Nevertheless, most studies have so far showed a scarce adoption of social networks by health-care organizations, mainly due to a poor managerial culture, which is many because practitioners do not recognize their potentialities, e.g., to reinforce the relationship with patients and improve the reputation of the organization [10,45,46]. Even those that are currently using social networks (mainly Facebook or Twitter) do not fully exploit them to create positive interactions with their patients and dialogically communicate about health-related themes with followers. Social networks are used to distribute information (e.g., news and events about the hospital), rather than capitalizing on the interactivity enabled by a constellation of tools and technologies that support peer-to-peer conversations among the health-care organizations, patients, and all stakeholders, crucial to engage and maintain a relationship with them [45,46].

The challenge for health-care providers is to understand where the conversation is already being held and intervene to direct it in the desired direction [4,47]. Several studies have underlined the importance that health-care organizations elaborate a strategic plan for digital communication centred around informational as well as emotionally focused conversations [3,42,48] that consider the specificities of the health-care consumers, who usually are more sensitive to potential risks associated with health-related information-seeking and sharing behaviours on social networks [48,49,50]. Health-care staff actively contribute to this conversation so that the inappropriate use of social networks (e.g., complaints about patients which evidence, even if anonymous, a lack of empathy and respect) may damage the organization and raise legal and/or ethical issues with negative consequences for organizational reputation [2,51].

From an operational point of view, a team of professionals should be devoted to the digital communications, i.e., to keep the social networks’ page dynamic, attractive as well as updated and interactive with frequent and co-shared posts in order to build and maintain relationships with patients and followers [42,46]. The demanding task is stimulating the participation of followers based on the credibility and usefulness of health information provided on the page [48] and/or leveraging “opinion leader”, i.e., public health influencers driving online conversation on health [3,52]. If, at the beginning, it is quite manageable to get the first impulse for social networks’ pages, their maintenance is not so simple. Indeed, it is necessary to keep the page alive, monitor information for quality and reliability, minimize the privacy risks, respond timely to questions or comments received via social media channels, and create opportunities for users to engage with the organization and with each other [3,4,43,48].

#### 3.2.2. Health-Care Professionals

A variety of motivations for health-care professionals (including physician, surgeons, psychologists, etc.) to use social networks have been reported in the literature. These include the opportunity to exploit these platforms for professional development, i.e., exchange of information and experiences among colleagues within community of practices [30,32,53,54]; for improving therapies by combining physical and online sessions [55] and for self-promoting interests to increase his/her own professional reputation and attract new patients [51,56,57].

Social networks have emerged as platforms for knowledge dissemination, exchange of medical information with peers, and interpersonal communication for healthcare professionals [53]. Clinicians/doctors share online treatments and therapies on patients and access data on similar situations (i.e., validation of experience, information, treatments) through colleagues included in their professional network usually organized around clinical speciality. They are available to share knowledge and information because in these virtual communities—structured as community of practices—it prevails a culture of altruism, trust, collectivism and reciprocity, as well as a respectful non-competitive environment [54]. This makes social networks a source of medical opinion that improve medical decision-making quality and support individual learning and development [30,32,54].

As for the specific field of counselling with psychologists, Kerr and Van Houten (2020) have investigated the use of digital tools (e.g., smartphones, tablets) and social media by a group of Australian therapists during in-presence sessions and during what is called online counselling, web counselling, or internet psychotherapy [55]. Their study has shown an ongoing transformation of therapeutic practices toward a “comprehensive therapy” where e-therapy is combined with face-to-face sessions, which lead the patients to perceive that therapy goes beyond 1-h consultation per week. Indeed, they have at their disposal, e.g., online programs to learn new skills (such as anxiety management skills); exercises to train at home with audio-recorded of in-session relaxation exercises led by the therapist; tools for sending synchronous and asynchronous messages to the therapist (e.g., WhatsApp and emails). As for social networks, they are used for non-intrusive connections through therapists’ curated tools and patients’ generated tools, including sharing posts, pictures, videos that support the patients in developing cognitive coping skills and driving positive behavioural changes. Therapists also leverage social networks to analyze the motivations to use (e.g., social pressure) and online self-representation of patients and thus evaluate their mental health [58].

Finally, Social networks, such as Facebook, Twitter, Instagram and Youtube have been also started to be used by professional for marketing activities [39]. Meng et al. (2021) have shown that physicians devote voluntary time to share online general and specific health knowledge (e.g., free health articles) to increase their online reputations and attract more patients for paid health services, thus gaining economic returns [57]. In this process, patient involvement is important to physicians, because high involved patients are willing to read, e.g., free articles, evaluate his/her expertise and competences, which influence their willingness to engage with the physician (e.g., paying for health consultations) and recommend to other users, family and friends. However, some studies have pointed at the extra-work placed on professionals, as well as at the risks associated with e-professionals. These studies have stressed the importance of maintaining professional boundaries, and thus clearly separate professional and personal accounts on social networks to avoid those online interactions that negatively affect their professional reputation and career [51,56].

### 3.3. Social Networks’ Users

#### 3.3.1. Ill Patients

Patient groups have benefited from the use of social networks for health purposes [11] which enable them to have interactions with health-care professionals; other patients, who offer narratives about their experiences; and caregivers, i.e., friends and family, who support patients mainly from an emotional point of view [29].

As to which categories of patients use more social networks, most studies have focused on young people and found that they choose the social networks (Facebook, Twitter, Instagram, YouTube) which they consider to be more beneficial to themselves, e.g., to reduce loneliness, exchange information and health advices with their physicians, find contents to positive entertainment [36,58,59,60].

The literature has identified two major motivations for ill people to use social networks. The first concerns the “informational support” and relates to getting information and increasing knowledge about one’s own disease and its therapies by sharing the experiences with other users [56,58]. Social network enables patients (mainly those with chronic illness and disability) to access, combine and contribute health information by sharing—through video and text—their experiences with other patients and their social circle (i.e., their families and friends) having the same illness [11,32]. In this regard, communities (e.g., groups focused on diabetes management on Facebook) have been shown to help to share specialized knowledge with peers, create a self-image representation of diabetics, and used Facebook as community mobilization tool to exert pressures on politicians [11].

The second motivation concerns the “social and emotional support” gained from peer-to-peer interactions, which have been emphasized as the most critical benefits of social media settings [12,56]. Digital environments facilitate the empathy with online peers where each person (ill person or his/her own family members) can access help from the virtual communities while controlling their level of disclosure of their identity and condition [12,59]. This online peer-to-peer support has been revealed to be crucial for individuals with serious mental illnesses such as schizophrenia, schizoaffective disorder, or bipolar disorder who are turning to self-forming online communities to talk about their illness experiences, seek advice and learn from and support each other [1]. For young people, it has been showed that social networks may be the channel through which they publicly express their discomfort and depression as emerged in Moreno et al. (2011) who have found several posts on Facebook profile of college students referring explicitly to depressed mood (i.e., angry, empty, crying), decreased interest and pleasure in doing something, loss of energy, etc. [36].

However, according to Liu et al. (2020), these categories of support are strictly interrelated. Indeed, informational, emotional, esteem, and companionship supports positively influence members’ belongingness to online health communities, which affects information sharing, responsible, feedback, and advocacy behaviours [61]. The greater the sense of belongingness to an online community, the more people—even those with chronic or dependent illnesses—are willing to help others in the same situation by sharing experiences and by offering advice and suggestions to health providers.

#### 3.3.2. General Users

Social networks are used by public for obtaining information about healthy lifestyle, general understanding of health conditions and symptoms, side effects of medications, minor health issues having no social stigma (i.e., diseases people do not want to share publicly), as well as, to a lesser extent, for rating health-care providers [12,48,62,63]. Even though users look frequently for information on health communities and/or social networks’ pages of health-care providers, Thackeray et al. (2013) have noted that they are less likely to contribute by commenting or creating posts [64].

Some studies have underlined differences in using social networks depending on the users’ age. Old people use regularly social networks to complement information given by physicians, for finding about doctors’ experiences and career, for discovering potential therapies [65]. In contrast, young people are prolific users of social networks for materials on healthy lifestyle, i.e., physical activity, diet/nutrition and body image. Goodyears et al. (2019) distinguish five typologies of contents, i.e., (1) automatically sourced contents promoted to young people (Instagram section “search and explore”) based on their likes, followers, etc.; (2) recommended health-related content, i.e., contents on specific health-related information deliberately searched by young people; (3) Peer contents, created by other young people (e.g., selfies) which encouraged bodies comparison; (4) Likes, which include the affirmation and endorsement of health-related information; (5) reputable contents published by official organisations, sports men and women, commercial brands [66]. Nevertheless, both groups of users have expressed concerns in terms of abundance and redundancy of information, lack of guarantee of professionalism, privacy and psychological risks for vulnerable people [48,50,65].

The studies reported in the literature have also addressed distinct process of appropriation of social networks. Some people use Instagram for self-representing a healthy identity. In so doing, they are subject to the persistent pressures from self and from the community. Both impose to perform the identity of being a healthy role model, with the following development of compulsions to use technologies to document and share many aspects of health and lifestyle, in which they remain blocked also when they attempt to disengage and detox temporarily or permanently [67]. Others use Facebook for acting as an opinion leader in their circle of virtual friends and, thus, advertising weight-loss products through news feeds and walls posting [68]. Interestingly, the more they are perceived to be benevolent, the more they obtain their followers purchase a sponsored weight-loss product after seeing their advertisement. Finally, Hacker et al. (2017) have evidenced that some people use a specific application on Facebook (i.e., Crunch calories) to get the support from their friends (i.e., social capital) to engage in health behaviours (diet and physical activity) [8,69,70]. 

## 4. Envisaging Fruitful Directions for Future Research

This scoping review has provided evidence that social networks may create a place to share, comment, and discuss information on a diverse range of health-related issues [4]. This is a persistent online place for messages sent by a variety of actors and digital communication unfolds as an ongoing process because these platforms are persistently available whether or not a user is active, and users can access wherever they are [71,72].

However, several gaps in the literature have been identified that need to be addressed by researchers to enrich existing literature and provide practitioners with guidelines about how to optimize the use of social networks in healthcare. From a methodological point of view, further research with larger sample size and robust methodologies are required in longitudinal qualitative e quantitative studies to understand how the digital communication changes and evolves over time.

Then, understanding the objectives and motivations of health-care providers and users in using social networks is important, but it is also urgent to dwell on the “process” in order to clarify the distinct processes of appropriation of social networks by different actors using these web-based technologies for health-care purposes.

As for users, Kietzmann et al. (2011) have identified a number of functional building blocks of social networks [73]. In details, social networks may be used by users to reveal themselves (“identity”), to show others to be available (“presence”), to exchange distribute and receive contents (“sharing”), to communicate with others (“conversation”), to know others social standing (“reputation”), to relate with each other (“relationships”) and form communities (“groups”). Future studies could build on these conceptual categories and investigate the distinct processes of appropriation of social networks by users for each of the identified functionalities.

Likewise, further studies are needed to understand the process of appropriation of social networks by health-care organizations and implications for their relationships with users. For example, hospitals have stable information for which continuous updates on social networks (e.g., schedules, professionals, departments) are not necessary. Therefore, it is necessary to investigate which ad hoc contents (e.g., scientific, institutional, health-related information) and social networks (e.g., videos on YouTube) enable both public and private hospitals to capitalize on social media’s potentialities, thus engaging users and positively influencing the image and reputation of the hospital. These investigations should include the specific context in which health-care organizations are embedded and engaged. We refer, e.g., to the geographical dimensions, because the cultural characteristics of the area where the hospital is located influence the type of language to be used. Another key contextual factor to consider concerns the public and private nature of hospitals. In fact, public hospitals reflect a single health-care system, while private hospitals live a competitive dynamic with other private facilities and with public healthcare and may decide to leverage digital communication to differentiate themselves from competitors.

Finally, considered the recent epidemiological emergency, future research should be directed at understanding how governments have used social networks during the pandemic, e.g., Presidents of some countries, including Italy, used also Facebook platforms to broadcast press conferences with updates on the epidemiological emergency to the whole community. Likewise, from the perspective of users, it is important to understand, e.g., the significance of these official communications and their role in raising awareness of the virus and the importance of adhering to rules and behaviours to avoid infection.

## 5. Conclusions

This comprehensive review has broken down literature on social networks’ use in the health-care industry into three distinct level of analysis, which reflect the viewpoints of the major actors using social networks for health-care purposes, i.e., governments, health-care providers (including health-care organizations and professionals) and social networks’ users (including ill patients and general public). While the first group of studies have addressed the social opportunities, along with the digital and privacy issues following the diffusion of social networks with health-related purposes, the group of studies on health-care organizations has mainly analysed the organizational drivers and barriers to the adoption of digital communication as well as attempted to understand the main assumptions to elaborate a strategic plan for an effective digital communication by health-care organizations. Much of the remaining literature is focused on attitudes and motivations for professionals and users to use social networks for health-related purposes. Health-care professionals may be motivated by moral reasons, i.e., sharing experiences and best practices for professional development and improved health outcomes, and in other cases they may be motivated by purely opportunistic and hedonistic reasons aimed at improving their online reputation to attract new patients. Clearly, one is not mutually exclusive, as for some medical categories, such as the beauty industry, it is becoming essential to use social channels to promote their beauty treatments and services. Different motivations drive patients and general users of social networks. In fact, while the formers seek to obtain information on their illnesses and above all to have the comfort of people who are living the health condition, the latter use social networks to publicly transmit a precise image of themselves that reflects the adoption of healthy lifestyles, or to get support from friends to engage in health behaviours.

Although these studies provide useful indications for understanding the use of social networks in healthcare, there are still many questions to be analysed, taking into account the rapid change that affects two interconnected levels. We refer, on the one hand, to the continuous and rapid diffusion of new social networking platforms and, on the other hand, to their use with new purposes and modalities often not as a consequence of a specific design of their creators but rather as a consequence of the way in which users appropriate these tools.

## Figures and Tables

**Table 1:** Distribution of documents for level of analysis.

Level of Analysis		References	Main Themes
Governments		Belfiore et al. (2020); Burton-Jones et al. (2020); Centola (2013); Chou et al. (2018); Dredze (2012); Goh et al. (2016); Griffiths et al. (2012); Gruzd and Mai (2020); Lau et al. (2012); Moreno et al. (2011); Munson et al. (2013); Nguyen et al. (2020); Norman (2012); Priya and Devapriya (2019); Williams (2010)	Opportunities arising from the use of social networks: - Reduction in social inequalities; - Exercise bio-surveillance; - Promotion of healthy lifestyles. Challenges arising from the use of social networks: - Solve digital divide; - Address misinformation; - Privacy issues with personal health information.
Health-care providers	*Health-care organizations*	Avery et al. (2010); Cain (2011); Gonçalves, G. (2020); Griffith et al. (2012); Heldman et al. (2013); Korda and Itani (2013); Li et al. 2018; Miller and Tucker 2013; Moorhead et al. (2013); Neiger et al. 2012; Nobre et al. (2019); Pinto (2015); Sarasohn-Kahn (2008); Thackeray (2012); Ventola (2014); Zhang et al. (2017)	- Drivers and barriers to the adoption of social networks in health-care organizations; - Strategic plans for digital communication in health-care organizations.
	*Health-care professionals*	Antheunis et al. (2013); Cain (2011); Griffith et al. (2012); Hazzam and Lahrech (2018); Jattamart and Leelasantitham (2020); Kerr and Van Houten (2020); Meng et al. (2021); Razzaque et al. (2013); Rolls et al. (2016)	*Motivations of professionals in using social networks:* - For knowledge sharing with colleagues (i.e., community of practice); - For improving therapies by combining physical and online sessions; - For self-promoting interests to attract new patients.
Social network’s users	*Patients*	Antheunis et al. (2013); Chou et al. (2009); Griffith et al. (2012); Househ et al. (2014); Liu et al. (2020); Moreno et al. (2011); Munson et al. (2013); Naslund et al. (2016); Rozental et al. (2010); Zhao and Zhang (2017)	*Motivations of patients in using social networks:* - Obtain informational support; - Obtain emotional (peer-to-peer) support.
	*General users*	Centola (2013); De Choudhury et al. (2014); Goodyears et al. (2019); Hacker et al. (2017); Kent (2020); Li et al. (2018); Maloney et al. (2014); Raghupathi and Fogel (2013); Sanders et al. (2015); Thackeray et al. (2013); Van de Belt et al. (2013); Zhang et al. (2017)	- Motivations and main concerns of users when search/share health-related information on social networks; - Appropriation of social networks for health-related purposes.

Source: our elaboration.

**Table 2:** Distribution of studies by methodology.

Methodology	Percentage of Studies
Survey (17)	53%
Case studies (2)	8%
Other secondary data (e.g., internet search) (10)	30%
Qualitative technique (e.g., interviews, focus group) (2)	5%
Multi-method research (1)	2%
Other (1)	2%
Total (33)	100

Source: our elaboration.

## References

[1] Naslund J.A., Aschbrenner K.A., Marsch L.A., Bartels S.J. (2016). The future of mental health care: Peer-to-peer support and social media. Epidemiol. Psychiatr. Sci..

[2] Ventola C.L. (2014). Social media and health care professionals: Benefits, risks, and best practices. Pharm. Ther..

[3] Korda H., Itani Z. (2013). Harnessing Social Media for Health Promotion and Behavior Change. Health Promot. Pract..

[4] Moorhead S.A., Hazlett D.E., Harrison L., Carroll J.K., Irwin A., Hoving C. (2013). A New Dimension of Health Care: Systematic Review of the Uses, Benefits, and Limitations of Social Media for Health Communication. J. Med. Internet Res..

[5] Centola D. (2013). Social Media and the Science of Health Behavior. Circulation.

[6] Belfiore P., Scaletti A., Frau A., Ripani M., Spica V.R., Liguori G. (2018). Economic aspects and managerial implications of the new technology in the treatment of low back pain. Technol. Health Care.

[7] Belfiore P., Sarnacchiaro P., Sorrentini A., Ricchiuti R. (2020). New social media for health promotion management: A statistical analysis. Soft Comput..

[8] Vaccarezza M., Papa V., Gemmati D., Tisato V. (2020). Sex/Gender Imbalance in CVD: Could Physical Activity Help to Improve Clinical Outcome Targeting CVD Molecular Mechanism in Women?. Int. J. Mol. Sci..

[9] Banegas J.R., Cabrera R.H., García E.L., Pérez-Regadera A.G., Rodríguez-Artalejo F. (2005). Social network and health-related quality of life in older adults: A population-based study in Spain. Qual. Life Res..

[10] Avery E., Lariscy R., Amador E., Ickowitz T., Primm C., Taylor A. (2010). Diffusion of Social Media among Public Relations Practitioners in Health Departments across Various Community Population Sizes. J. Public Relat. Res..

[11] Househ M., Borycki E., Kushniruk A. (2014). Empowering patients through social media: The benefits and challenges. Health Inform. J..

[12] Zhao Y., Zhang J. (2017). Consumer health information seeking in social media: A literature review. Health Inf. Libr. J..

[13] Spanò R., Ferri L. (2020). Information Systems in healthcare. Current Issues and Future Trends.

[14] Arksey H., O’Malley L. (2005). Scoping studies: Towards a methodological framework. Int. J. Soc. Res. Methodol..

[15] Daudt H.M., van Mossel C., Scott S.J. (2013). Enhancing the scoping study methodology: A large, inter-professional team’s experience with Arksey and O’Malley’s framework. BMC Med. Res. Methodol..

[16] Denyer D., Tranfield D., Buchanan D., Bryman A. (2009). Producing a systematic review. The Sage Handbook of Organizational Research Methods.

[17] Pham M.T., Rajić A., Greig J.D., Sargeant J.M., Papadopoulos A., McEwen S.A. (2014). A scoping review of scoping reviews: Advancing the approach and enhancing the consistency. Res. Synth. Methods.

[18] Snyder H. (2019). Literature review as a research methodology: An overview and guidelines. J. Bus. Res..

[19] Tranfield D., Denyer D., Smart P. (2003). Towards a Methodology for Developing Evidence-Informed Management Knowledge by Means of Systematic Review. Br. J. Manag..

[20] Tricco A.C., Lillie E., Zarin W., O’Brien K., Colquhoun H., Kastner M., Levac D., Ng C., Sharpe J.P., Wilson K. (2016). A scoping review on the conduct and reporting of scoping reviews. BMC Med. Res. Methodol..

[21] Webster J., Watson R.T. (2002). Analyzing the past to prepare for the future: Writing a literature review. MIS Q..

[22] Page M.J., McKenzie J.E., Bossuyt P.M., Boutron I., Hoffmann T.C., Mulrow C.D., Shamseer L., Tetzlaff J.M., Akl E.A., Brennan S.E. (2021). The PRISMA 2020 statement: An updated guideline for reporting systematic reviews. BMJ.

[23] Levine-Clark M., Esther L.G. (2008). A Comparative Citation Analysis of Web of Science, Scopus, and Google Scholar. J. Bus. Financ. Librariansh..

[24] Brien S.E., Lorenzetti D.L., Lewis S., Kennedy J., Ghali W.A. (2010). Overview of a formal scoping review on health system report cards. Implement. Sci..

[25] Thorpe R., Holt R., MacPherson A., Pittaway L. (2005). Using knowledge within small and medium-sized firms: A systematic review of the evidence. Int. J. Manag. Rev..

[26] Torraco R.J. (2016). Writing integrative literature reviews: Using the past and present to explore the future. Hum. Resour. Dev. Rev..

[27] Sandelowski M., Barroso J. (2006). Handbook for Synthesizing Qualitative Research.

[28] Burton-Jones A., Akhlaghpour S., Ayre S., Barde P., Staib A., Sullivan C. (2020). Changing the conversation on evaluating digital transformation in healthcare: Insights from an institutional analysis. Inf. Organ..

[29] Munson S.A., Cavusoglu H., Frisch L., Fels S. (2013). Sociotechnical Challenges and Progress in Using Social Media for Health. J. Med. Internet Res..

[30] Razzaque A., Eldabi T., Jalal-Karim A. (2013). Physician virtual community and medical decision making: Mediating role of knowledge sharing. J. Enterp. Inf. Manag..

[31] Goh J.M., Gao G., Agarwal R. (2016). The Creation of Social Value: Can an Online Health Community Reduce Rural-Urban Health Disparities?. MIS Q..

[32] Griffiths F., Cave J., Boardman F., Ren J., Pawlikowska T., Ball R., Clarke A., Cohen A. (2012). Social networks—The future for health care delivery. Soc. Sci. Med..

[33] Norman C.D. (2012). Social media and health promotion. Glob. Health Promot..

[34] Dredze M. (2012). How Social Media Will Change Public Health. IEEE Intell. Syst..

[35] Priya R.S., Devapriya M. (2019). The role of pre-processing on unstructured and informal text in diabetic drug related twitter data. Int. J. Sci. Technol. Res..

[36] Moreno M.A., Jelenchick L.A., Egan K.G., Cox E., Young H., Gannon K.E., Becker T. (2011). Feeling bad on Facebook: Depression disclosures by college students on a social networking site. Depress. Anxiety.

[37] Gruzd A., Mai P. (2020). Going viral: How a single tweet spawned a COVID-19 conspiracy theory on Twitter. Big Data Soc..

[38] Lau A.Y., Gabarron E., Fernandez-Luque L., Armayones M. (2012). Social Media in Health—What are the Safety Concerns for Health Consumers?. Health Inf. Manag. J..

[39] Williams J. Social networking applications in health care: Threats to the privacy and security of health information. Proceedings of the 2010 ICSE Workshop on Software Engineering in Health Care.

[40] Nguyen M.H., Gruber J., Fuchs J., Marler W., Hunsaker A., Hargittai E. (2020). Changes in Digital Communication during the COVID-19 Global Pandemic: Implications for Digital Inequality and Future Research. Soc. Media Soc..

[41] Chou W.-Y.S., Oh A., Klein W.M.P. (2018). Addressing Health-Related Misinformation on Social Media. JAMA.

[42] Heldman A.B., Schindelar J., Weaver J.B. (2013). Social Media Engagement and Public Health Communication: Implications for Public Health Organizations Being Truly “Social”. Public Health Rev..

[43] Nobre H., Szczygiel N., Condé-Pinto M. (2019). Communicating with patients through Facebook: The case of dental healthcare services. Int. J. Bus. Excell..

[44] Pinto M.B. (2015). Social media’s contribution to customer satisfaction with services. Serv. Ind. J..

[45] Thackeray R., Neiger B.L., Smith A.K., Van Wagenen S.B. (2012). Adoption and use of social media among public health departments. BMC Public Health.

[46] Gonçalves G. (2020). Are hospitals our friends? An exploratory study on the role of Facebook in hospital organizations’ dialogic communication. Health Mark. Q..

[47] Neiger B.L., Thackeray R., Van Wagenen S.A., Hanson C.L., West J.H., Barnes M.D., Fagen M.C. (2012). Use of social media in health promotion: Purposes, key performance indicators, and evaluation metrics. Health Promot. Pract..

[48] Li Y., Wang X., Lin X., Hajli M. (2018). Seeking and sharing health information on social media: A net valence model and cross-cultural comparison. Technol. Forecast. Soc. Chang..

[49] Miller A.R., Tucker C. (2013). Active social media management: The case of health care. Inf. Syst. Res..

[50] Zhang X., Wen D., Liang J., Lei J. (2017). How the public uses social media wechat to obtain health information in China: A survey study. BMC Med. Inform. Decis. Mak..

[51] Cain J. (2011). Social media in health care: The case for organizational policy and employee education. Am. J. Health Pharm..

[52] Sarasohn-Kahn J. (2008). The Wisdom of Patients: Health Care Meets Online Social Media.

[53] Hazzam J., Lahrech A. (2018). Health Care Professionals’ Social Media Behavior and the Underlying Factors of Social Media Adoption and Use: Quantitative Study. J. Med. Internet Res..

[54] Rolls K., Hansen M., Jackson D., Elliott D. (2016). How Health Care Professionals Use Social Media to Create Virtual Communities: An Integrative Review. J. Med. Internet Res..

[55] Kerr J., Van Houten A. (2020). Utilizing digital tools to support face-to-face care: Examining uptake within the practices of Australian psychologists. Hum. Technol..

[56] Antheunis M.L., Tates K., Nieboer T. (2013). Patients’ and health professionals’ use of social media in health care: Motives, barriers and expectations. Patient Educ. Couns..

[57] Meng F., Zhang X., Liu L., Ren C. (2021). Converting readers to patients? From free to paid knowledge-sharing in online health communities. Inf. Process. Manag..

[58] Jattamart A., Leelasantitham A. (2020). Perspectives to social media usage of depressed patients and caregivers affecting to change the health behavior of patients in terms of information and perceived privacy risks. Heliyon.

[59] Chou W.Y.S., Hunt Y.M., Beckjord E.B., Moser R.P., Hesse B.W. (2009). Social media use in the United States: Implications for health communication. J. Med. Internet Res..

[60] Rozental T.D., George T.M., Chacko A.T. (2010). Social Networking among Upper Extremity Patients. J. Hand Surg..

[61] Liu S., Xiao W., Fang C., Zhang X., Lin J. (2020). Social support, belongingness, and value co-creation behaviors in online health communities. Telemat. Inform..

[62] De Choudhury M., Morris M.R., White R.W. Seeking and sharing health information online: Comparing search engines and social media. Proceedings of the SIGCHI Conference on Human Factors in Computing Systems.

[63] Van de Belt T.H., Engelen L.J., Berben S.A., Teerenstra S., Samsom M., Schoonhoven L. (2013). Internet and Social Media for Health-Related Information and Communication in Health Care: Preferences of the Dutch General Population. J. Med. Internet Res..

[64] Thackeray R., Crookston B., West J.H. (2013). Correlates of Health-Related Social Media Use among Adults. J. Med. Internet Res..

[65] Sanders K., Valle M.S., Viñarás M., Llorente C. (2015). Do we trust and are we empowered by “Dr. Google”? Older Spaniards’ uses and views of digital healthcare communication. Public Relat. Rev..

[66] Goodyear V.A., Armour K.M., Wood H. (2019). Young people and their engagement with health-related social media: New perspectives. Sport Educ. Soc..

[67] Kent R. (2020). Self-Tracking Health over Time: From the Use of Instagram to Perform Optimal Health to the Protective Shield of the Digital Detox. Soc. Media Soc..

[68] Raghupathi V., Fogel J. (2013). Facebook advertisements and purchase of weight-loss products. J. Med. Mark..

[69] Hacker J., Wickramasinghe N., Durst C. (2017). Can health 2.0 address critical healthcare challenges? Insights from the case of how online social networks can assist in combatting the obesity epidemic. Australas. J. Inf. Syst..

[70] Maloney S., Moss A., Ilic D. (2014). Social media in health professional education: A student perspective on user levels and prospective applications. Adv. Health Sci. Educ..

[71] Pianese T. Social network in sports events: A process study of the 30th Summer Universiade. Proceedings of the Euram 2021 Conference “Reshaping Capitalism for a Sustainable World”.

[72] MacNamara J., Zerfass A. (2012). Social Media Communication in Organizations: The Challenges of Balancing Openness, Strategy, and Management. Int. J. Strat. Commun..

[73] Kietzmann J.H., Hermkens K., McCarthy I.P., Silvestre B.S. (2011). Social media? Get serious! Understanding the functional building blocks of social media. Bus. Horiz..

